# Intraductal Papillary Mucinous Neoplasm of the Pancreas: Understanding the Basics and Beyond

**DOI:** 10.7759/cureus.3867

**Published:** 2019-01-11

**Authors:** Simcha Weissman, Rishi Thaker, Noam Zeffren, Rayan Sarfaraz, John Dedousis

**Affiliations:** 1 Internal Medicine, Touro College of Osteopathic Medicine, New York, USA; 2 Internal Medicine, Hoboken University Medical Center, Hoboken, USA; 3 Internal Medicine, Bayonne Medical Center, Bayonne, USA

**Keywords:** impns, pancreatic cystic lesion, gastrointestinal endoscopy

## Abstract

Intraductal papillary mucinous neoplasm (IPMN) is a benign cystic lesion that grows in the pancreatic ductal system. While the risk for undergoing malignant transformation is dependent on a number of factors, the risk is certainly present, differentiating it from other cystic lesions of the pancreas. Additionally, IMPN is to be starkly contrasted with adenocarcinoma of the pancreas, which is by nature malignant. There are numerous ways to detect IPMN, which is helpful, as a patient may be initially asymptomatic at presentation. Prognosis varies depending upon the malignant potential of the lesion at hand. Surgical resection is the mainstay of treatment in patients with a high probability of malignancy potential. What once was a very confusing diagnosis is now becoming defined based on new literature. The goal of this manuscript is to compile the literature on IPMNs in a clear and precise way as to educate clinicians as to the nature of this increasingly prevalent disease.

## Introduction and background

**Figure 1:**
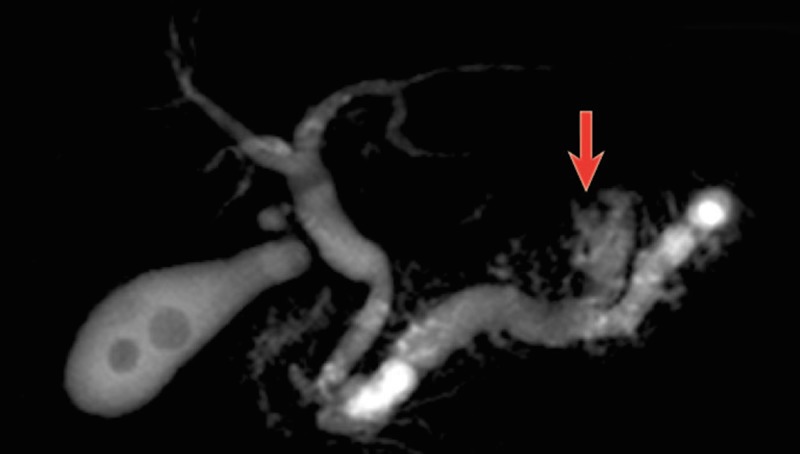
MRCP of the pancreatic ductal system showing a branch-duct IPMN illustrated by the arrow [5]; MRCP: magnetic resonance cholangiopancreatography, IPMN: intraductal papillary mucinous neoplasm

**Figure 2:**
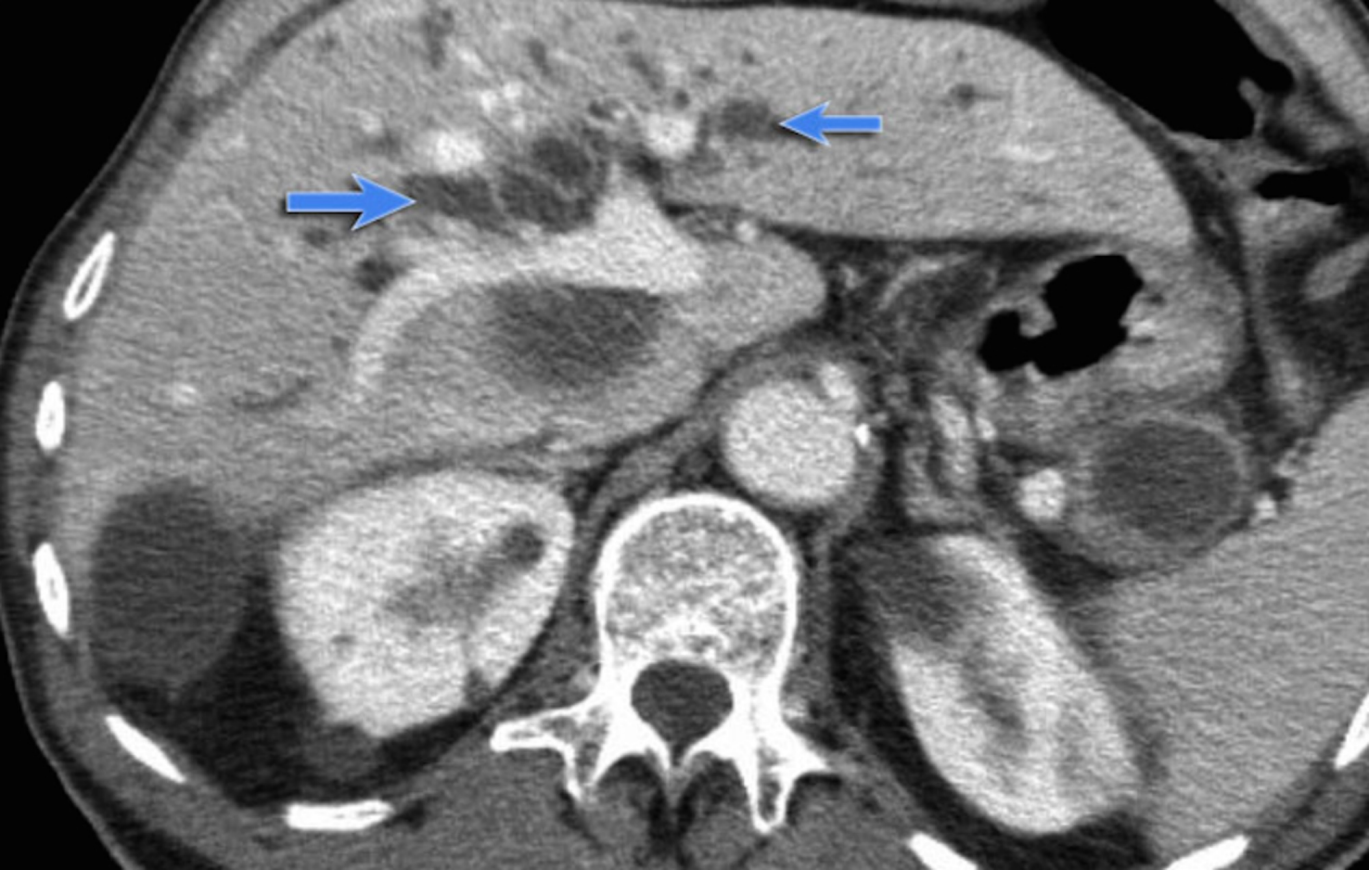
CT scan of the abdomen showing a main-duct IPMN indicated by arrows [5]; CT: computed tomography, IPMN: intraductal papillary mucinous neoplasm

**Figure 3:**
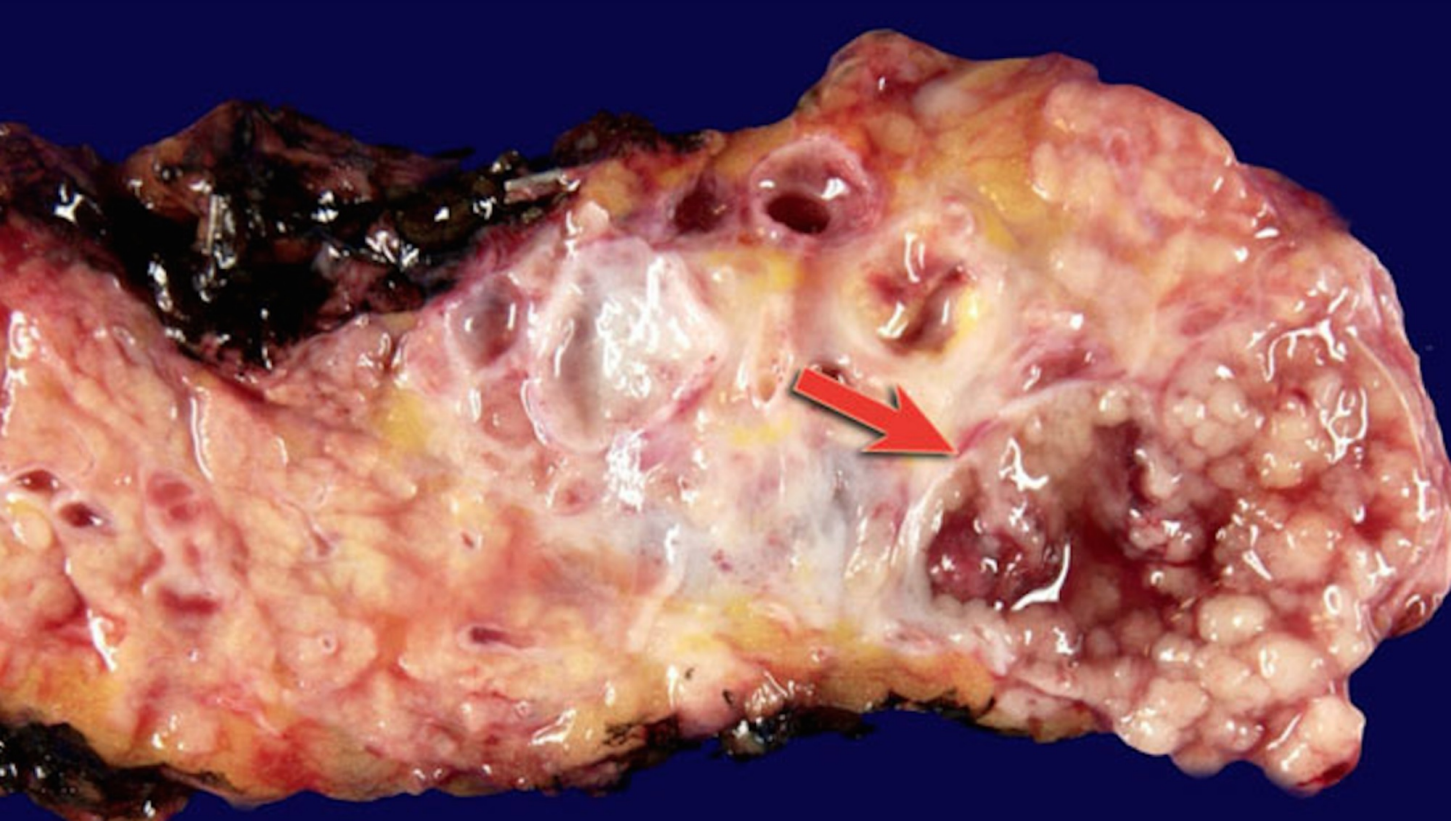
Macroscopic specimen of the pancreas depicting an IPMN with an arrow illustrating the extensive mucinous production from within the body of the cystic tumor [5]; IPMN: intraductal papillary mucinous neoplasm

Intraductal papillary mucinous neoplasm (IPMN) of the pancreas is defined as cystic papillary growths within the pancreatic ductal system, capable of secreting thick mucinous materials. IPMNs were first reported in the 1970s but only became recognized as the unique pancreatic neoplasms we know them as today in the 1990s [1]. IPMNs are becoming more routinely diagnosed due to recent advances in diagnostic procedures and imaging techniques. There are several types of IPMN due to the precise location they present. A type I lesion occurs in the main duct, a type II lesion in the branch ducts, and a type III lesion, the mixed type, occurs in both the main and branch ducts of the pancreatic ductal system (Figures 1-2). There has been no established etiology to the pathogenesis of IMPN nor has there been any linkage to genetic or familial entities. One feature that has been concrete is their ability to secrete an enormous amount of mucinous material (Figure 3). This is important as leaking mucous from the ampulla of vater—better known as the hepatopancreatic ampulla or duct—can at times be visualized on gastrointestinal endoscopy. Another tendency of these lesions is their relatively slow growth, as the natural progression of an IPMN to cancer is approximately five years [2]. According to a case series in 2004, IPMNs accounted for approximately 2% of exocrine pancreatic neoplasms and almost 20% of cystic pancreatic neoplasms [3]. According to another study, perhaps as many as 50% of cystic lesions found in the pancreas were IPMNs [4]. Other cystic lesions of the pancreas include mucinous cystadenomas, serous cystadenomas, pancreatic pseudocysts, and pseudopapillary neoplasms. These lesions have a very low malignant potential with a relatively favorable prognosis. What makes IMPN of particular interest is their capability of becoming malignant combined with their unique ability to be resected before cancer becomes a true clinical phenomenon. 

## Review

IPMNs of the pancreas are becoming increasingly popular in literature as well as in their disease prevalence. The fact that they are pre-cancerous lesions is important, as resection allows for a termination to cancer progression. As adenocarcinoma of the pancreas has a mortality rate approaching 80% to 90%, the distinction between these two lesions is of paramount importance. On the same note, some of the other cystic pancreatic lesions have very little activity in the way of malignancy potential.

Disease prevalence 

While pancreatic cysts, in general, are present in about 2.5% of patients, more than 95% of these lesions are benign [6]. However, it can be as high as 10% of people over the age of 70 years. By contrast, the prevalence of persons with IPMN is approximately 0.001% to 0.002% [7]. Interestingly, approximately 40% to 65% of IPMNs occur in the branch ducts of the pancreas, while they are only found in the main duct in 15% to 35% of cases and found combined only in 15% to 20% of cases [8]. Compared to main-duct IPMN (MD-IPMN), branch-duct IPMN (BD-IPMN) is more commonly found in females, 57% and 55.5% respectively. IPMNs were also found more frequently in the proximal pancreas as opposed to the tail [9].

Evaluating malignancy potential

The location of lesion origin is a significant factor in determining malignancy potential. The frequency of malignancy in main duct lesions is close to 80% [10]. By relatively stark contrast, the frequency of malignancy in branch duct lesions is significantly lower, with an average of around 20% [11]. According to some studies, the presence of a mural nodule during endoscopic ultrasound (EUS) is the most reliable indicator for malignancy potential and should thus be surgically resected indefinitely [12]. IPMN size >3 cm was once thought to be among the leading predictors of malignancy transformation. However, it is now understood that size alone is not a significant predictor of malignancy in MD-IPMN, BD-IMPN, or mixed-type IPMNs [13]. IPMNs less than 10 mm in size may still confer a risk of malignancy transformation [14]. Although size is an unreliable predictor of malignancy, patients with large lesions should be monitored even more closely [15].

Signs and symptoms

Clinical symptoms of undiagnosed IPMN and its subtypes can often be composed of masked or nonspecific complaints [16]. Although many of its symptoms overlap with other pancreatic as well as biliary tree pathology, there are few, if any, specific symptoms that point discriminately to a mucinous neoplasm of the pancreas. Possible symptoms include vague right quadrant or epigastric abdominal pain, abdominal distension, pain radiating to the back, nausea, vomiting, jaundice, icteric sclera, fatigue, weight loss, tachycardia, hypotension, fever, pruritus, and anorexia. Worsening control or recent onset of glucose intolerance can be another manifestation. However, of clinical importance, patients may not exhibit any symptoms whatsoever [1,8,17-18].

Diagnostics

The diagnosis of IPMNs involves several possible methods of testing. Although there is no current "gold standard" for diagnosis, diagnosis can be made using radiologic findings, endoscopic visualization, tumor markers, or evaluation of cytology. Abdominal computed tomography (CT) scan is the most widely used radiologic modality used for the diagnosis of IPMN. CT can provide imaging revealing lesions involving the pancreas, including pancreatic duct dilation associated with excess mucinous production, cystic lesions, parenchymal atrophy, and pancreatic masses. Endoscopic evaluation can be performed in symptomatic patients presenting with obstructive jaundice. Endoscopic retrograde cholangiopancreatography (ERCP) has been successfully used for early diagnosis. The pathognomonic finding on ERCP reveals an image of excessive mucinous secretion as well as swelling in the papilla along with progressive dilation of the main pancreatic duct. Magnetic resonance cholangiopancreatography (MRCP) is an alternative, non-invasive method of diagnosis with similar diagnostic accuracy as the ERCP. EUS is the preferred method of endoscopic evaluation as it allows for imaging, as well as intervention to perform fine needle aspiration (FNA) of cystic fluid for cytological examination. Cystic fluid can provide valuable tumor markers found in IPMN. It has been found that a carcinoembryonic antigen (CEA) level >192 in the cystic fluid is highly suggestive of a mucinous neoplasm. Extremely high levels of amylase in the cystic fluid, along with other modalities, should also be utilized for the diagnosis of IPMN [19]. 

Prognosis

According to a retrospective analysis of 15,269 pancreatic cancer cases in the California cancer registry, one-year and five-year survival rates for IPMNs were 83% and 65%, respectively, significantly higher than that of neuroendocrine tumors, mucinous tumors, or adenocarcinoma [20]. The malignant potential of IPMNs depends on its morphologic subtype. MD-IPMN has a transformation potential of 57% to 92%, BD-IPMN has a transformation potential of 6% to 46%, and mixed-type IPMNs occurring in both the main and branch ducts of the pancreatic ductal system have a variable malignant potential that new data suggests can range from 6% to 72% [21-23]. The true five-year survival of patients with IPMN depends on the existence of an invasive component. Non-invasive IPMNs (adenoma, borderline, or carcinoma in situ) had a five-year survival of 80% to 100%, while invasive IPMNs demonstrated a five-year survival of 40% to 60% [24]. Most of this difference in prognosis has to do with early detection and planned treatment. Recurrence rates for invasive IPMNs are as high as 67% according to one study of 113 surgical resections, and 91% happen within the first three years. Recurrence rates for non-invasive IPMNs were near zero and no difference was found between partial and total pancreatectomy for either invasive or non-invasive IPMNs [25].

Treatment

International consensus guidelines for the management of IPMNs indicate that MD-IPMNs should be resected by pancreaticoduodenectomy, total pancreatectomy, or partial pancreatectomy with lymph node dissection and clean margins, in all surgically fit patients due to their high malignant potential. BD-IPMNs should only be resected if they possess high-risk stigmata such as size >3 cm, clinical symptoms, or suspicious radiological findings such as mural nodules, main pancreatic and common bile duct dilation, rapidly increasing cyst size, or lymphadenopathy. For small BD-IPMNs that are candidates for longitudinal surveillance, EUS-FNA can be used to analyze cyst fluid and perform cytology to assess for high-grade epithelial atypia or genetic mutations at higher risk of malignant transformation. Mucosal ethanol ablation is still in the experimental phase of therapy, and while it may be promising, it is still not recommended therapy [26].

## Conclusions

In conclusion, while IPMNs have been an under-appreciated diagnosis for the past several decades, they are now known to be highly important. Their innate ability to become cancerous is perhaps what draws particular attention to this lesion. Among the common culprits of pancreatic cysts, they now remain the most clinically appreciated. While the diagnosis is much easier to make than ever before, differentiating them from other cystic lesions can still be challenging. With a high malignant potential in certain patients with an IMPN, rapid diagnosis and treatment are crucial for survival. Prognosis for post-surgical removal has proven to be promising as a mainstay of therapy. One should now understand the importance of such lesions, their presentation as well as how they should be appropriately managed. 
